# Light-focusing human micro-lenses generated from pluripotent stem cells model lens development and drug-induced cataract *in vitro*

**DOI:** 10.1242/dev.155838

**Published:** 2018-01-01

**Authors:** Patricia Murphy, Md Humayun Kabir, Tarini Srivastava, Michele E. Mason, Chitra U. Dewi, Seakcheng Lim, Andrian Yang, Djordje Djordjevic, Murray C. Killingsworth, Joshua W. K. Ho, David G. Harman, Michael D. O'Connor

**Affiliations:** 1 School of Medicine, Western Sydney University, Campbelltown, NSW 2560, Australia; 2 Medical Sciences Research Group, Western Sydney University, Campbelltown, NSW 2560, Australia; 3 Victor Chang Cardiac Research Institute, Darlinghurst, NSW 2010, Australia; 4 St Vincent's Clinical School, University of New South Wales, Sydney, NSW 2010, Australia; 5 Electron Microscopy Laboratory, NSW Health Pathology and Correlative Microscopy Facility, Ingham Institute, Liverpool, NSW 2170, Australia

**Keywords:** Lens development, Stem cell, Organoid, Focus, Cataract, Vx-770

## Abstract

Cataracts cause vision loss and blindness by impairing the ability of the ocular lens to focus light onto the retina. Various cataract risk factors have been identified, including drug treatments, age, smoking and diabetes. However, the molecular events responsible for these different forms of cataract are ill-defined, and the advent of modern cataract surgery in the 1960s virtually eliminated access to human lenses for research. Here, we demonstrate large-scale production of light-focusing human micro-lenses from spheroidal masses of human lens epithelial cells purified from differentiating pluripotent stem cells. The purified lens cells and micro-lenses display similar morphology, cellular arrangement, mRNA expression and protein expression to human lens cells and lenses. Exposing the micro-lenses to the emergent cystic fibrosis drug Vx-770 reduces micro-lens transparency and focusing ability. These human micro-lenses provide a powerful and large-scale platform for defining molecular disease mechanisms caused by cataract risk factors, for anti-cataract drug screening and for clinically relevant toxicity assays.

## Abstract (precis)

**Highlighted Article:** Using human pluripotent stem cells, robust and reliable methods are described for large-scale production of purified human lens epithelial cells, and for subsequent large-scale generation of clinically relevant, light-focusing micro-lenses.

## INTRODUCTION

During embryogenesis, the ocular lens arises from the lens placode in the surface ectoderm opposite the optic cup (Mann, 1964; Tholozan and Quinlan, 2007). Although the exact process can differ between vertebrate species, key lens features shared by vertebrates include an anterior lens epithelial cell (LEC) monolayer expressing α-crystallins overlying a mass of lens fibre cells expressing α-, β- and γ-crystallins (Thomson and Augusteyn, 1985). In mammals, invagination of the lens placode is followed by formation of the lens vesicle – a spherical LEC monolayer surrounding an acellular lumen. Differentiation of the posterior LECs into lens fibre cells fills the lens vesicle lumen to establish the basic lens architecture. For decades these features have provided a framework for *in vitro* lens and cataract studies using explanted primary rat LECs. For example, our group reported *in vitro* regeneration of light-focusing rat lenses from paired rat LEC monolayers arranged to mimic lens vesicles (O'Connor and McAvoy, 2007). The size, cellular arrangement and protein expression within these *in vitro* regenerated rat lenses closely resembled newborn rat lenses. Continued culture of these regenerated rat lenses resulted in formation of a human-like cataract, as seen by reduced light transmission and reduced focusing ability.

To improve the suitability of *in vitro* lens regeneration for targeted and large-scale cataract studies, we investigated human pluripotent stem cells (hPSCs) as a source of LECs. A handful of studies have differentiated hPSCs to relatively impure populations of lens cells or ‘lentoids’ – small aggregates of randomly organised LECs and lens fibre cells (Fu et al., 2017; Li et al., 2016; Yang et al., 2010). Limitations with these approaches include the presence of contaminating non-lens cells, the spontaneous and random nature of lentoid production, and the production of only tens-to-hundreds (Fu et al., 2017; Li et al., 2016) or thousands (Yang et al., 2010) of lentoids. Although one report describes limited magnification ability of the lentoids (Fu et al., 2017), none of the published methods have been shown to produce biconvex lentoids that focus light to a point – the fundamental functional requirement of the lens – due to abnormal attachment of the lentoids to culture surfaces and/or other cell types.

Here, we describe a simple and efficient system for production of 10^6^-10^8^ purified LECs from hPSCs, and the subsequent controlled, robust and reproducible production of 10^3^-10^5^ light-focusing human micro-lenses. These micro-lenses possess anatomical and molecular features of primary human lenses, and exposing the micro-lenses to the cystic fibrosis drug Vx-770 decreases their ability to transmit and focus light. This platform provides a robust and accessible human system for modelling lens and cataract development, anti-cataract drug screening, and drug toxicity studies.

## RESULTS

### Characterisation of ROR1 as a LEC marker

**Fig. 1.**
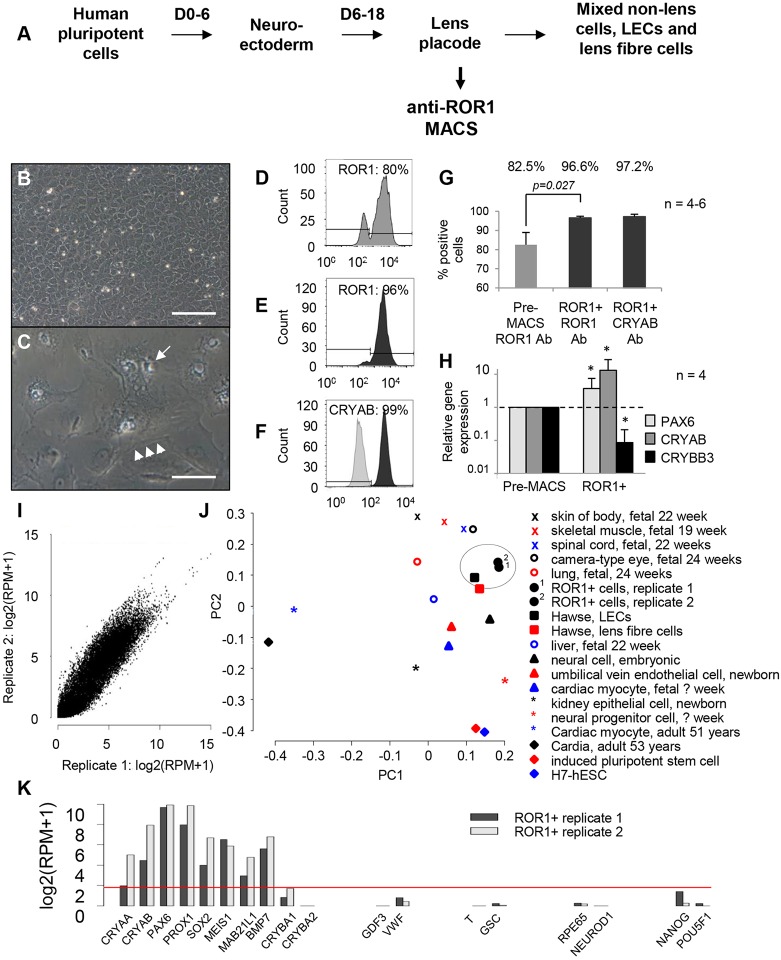
**Identification and characterisation of ROR1 as a LEC marker.** (A) Schematic diagram showing the three-stage lens differentiation protocol, with modification to enable ROR1-based purification of LECs. (B,C) ROR1^+^ cells cultured at high cell densities showed uniform polygonal morphologies that formed tightly packed monolayers (B). When cultured at low cell densities or passaged in medium containing only FGF2 (C), ROR1^+^ cells became large and vacuolated (arrow) with stress fibres (arrowheads; cells shown 18 days after plating; *n*=3). Scale bars: 100 µm. (D-G) Flow cytometry data showing expression levels of: ROR1 prior to (D) and after (E) ROR1-based purification; CRYAB after ROR1-based purification (F); and average expression levels before and after purification (G). (H) Relative mRNA transcript expression levels for PAX6, CRYAB and the lens fibre-specific gene *CRYBB3* after ROR1^+^ cell separation (*P<0.05). (I) Pearson correlation showing high similarity (>0.96) between RNA-seq libraries generated from two independent ROR1^+^ cell samples. (J) Principal component analysis shows the ROR1^+^ RNA-seq transcriptomes are most similar to primary human LECs (circled). (K) Representative data from the ROR1^+^ RNA-seq libraries shows key genes required by LECs are expressed (*CRYAA*, *CRYAB*, *PAX6*, *PROX1*, *SOX2*, *MEIS1*, *MAB21L1*, *BMP7*). In contrast, genes expressed by lens fibre cells (*CRYBA1*, *CRYBA2*) or various endodermal cells (*GDF3*, *VWF*), mesodermal cells (*T*, *GSC*), non-lens ectodermal cells (*RPE65*, *NEUROD1*) and pluripotent cells (*NANOG*, *POU5F1*) are not expressed. Data shown in B,C and D-H are representative of 50 and four (respectively) independent differentiation experiments using four different hPSC lines; data are mean±s.e.m. in G,H.

We hypothesised that the impurity of LECs generated from PSCs via published methods, together with suboptimal culture conditions for these LECs, leads to uncontrolled lentoid production, uncontrolled lentoid shape, random detachment and loss of lentoids from the culture, and the inability to focus light. By modifying (Fig. 1A) an elegant three-stage growth factor treatment for lens cell differentiation (Yang et al., 2010), we increased lentoid production, lentoid retention, and expression of LEC and lens fibre cell genes (Fig. S1). Nevertheless, heterogeneous cell morphologies were still obtained, lentoid production was still uncontrolled, lentoids still detached and were lost, and the lentoids did not focus light when assessed via light microscopy. As an alternative approach, analysis of published lens microarray data (Hawse et al., 2005) identified the receptor tyrosine kinase-like orphan receptor 1 (ROR1) as a potential LEC purification antigen (Fig. S2). *In situ* hybridisation showed ROR1 is highly expressed by mouse LECs at embryonic day 14, and PCR showed ROR1 transcript expression at a similar stage of the three-stage lens differentiation protocol.

Magnetic-activated cell sorting (MACS) using an anti-ROR1 antibody during stage 2 of the lens differentiation protocol (Fig. 1A) consistently produced a homogeneous population of polygonal cells (supplementary material File S2; Fig. 1B). However, this LEC-like morphology changed to large and vacuolated when the ROR1^+^ cells were cultured at low density or passaged in medium containing only FGF2 (Fig. 1C). These polygonal (LEC-like) and vacuolated (abnormal) cell morphologies are highly similar to primary human foetal LECs either when first explanted or when passaged (Ringens et al., 1982). Flow cytometry revealed that 95-100% of the captured cells were ROR1^+^, and over 99% expressed CRYAB (crystallin αB) (Fig. 1D-G). Comparing the ROR1^+^ cells with the starting population revealed that their transcriptional profile is consistent with purification of LECs. This included a threefold increase in PAX6, a 10-fold increase in CRYAB and a 10-fold decrease in CRYBB3 (crystallin βB3) expression (Fig. 1H). Comparing whole-transcriptome RNA-seq profiles from different ROR1^+^ cell batches showed highly similar expression patterns (Pearson correlation >0.96; Fig. 1I), indicating high reproducibility of the cell separation. Principal component analysis found the ROR1^+^ RNA-seq libraries to be most closely related to primary adult human LECs (Fig. 1J). This was further reinforced by gene set analysis where comparison of the ROR1^+^ RNA-seq data against a compendium of 145 human and 95 mouse gene expression datasets revealed the ROR1^+^ transcriptomes to be most similar to human LECs (Hawse et al., 2005) and mouse LECs (Hoang et al., 2014) (false discovery rates <9.6×10^−3^ and <5.88×10^−8^, respectively). Over 90 transcripts indicative of LECs (Lachke et al., 2012) were reproducibly expressed in the ROR1^+^ RNA-seq libraries, whereas genes associated with various endodermal, mesodermal, non-lens ectodermal or pluripotent cells were not (Fig. 1K), thus supporting the high purity and LEC nature of the ROR1^+^ cells. Consistent with this, transplantation of ROR1^+^ cells into immunocompromised mice showed no teratoma formation unless hPSCs were deliberately transplanted with the ROR1^+^ cells (Fig. S2).

### Combinatorial growth factor screening for ROR1^+^ proliferation

**Fig. 2.**
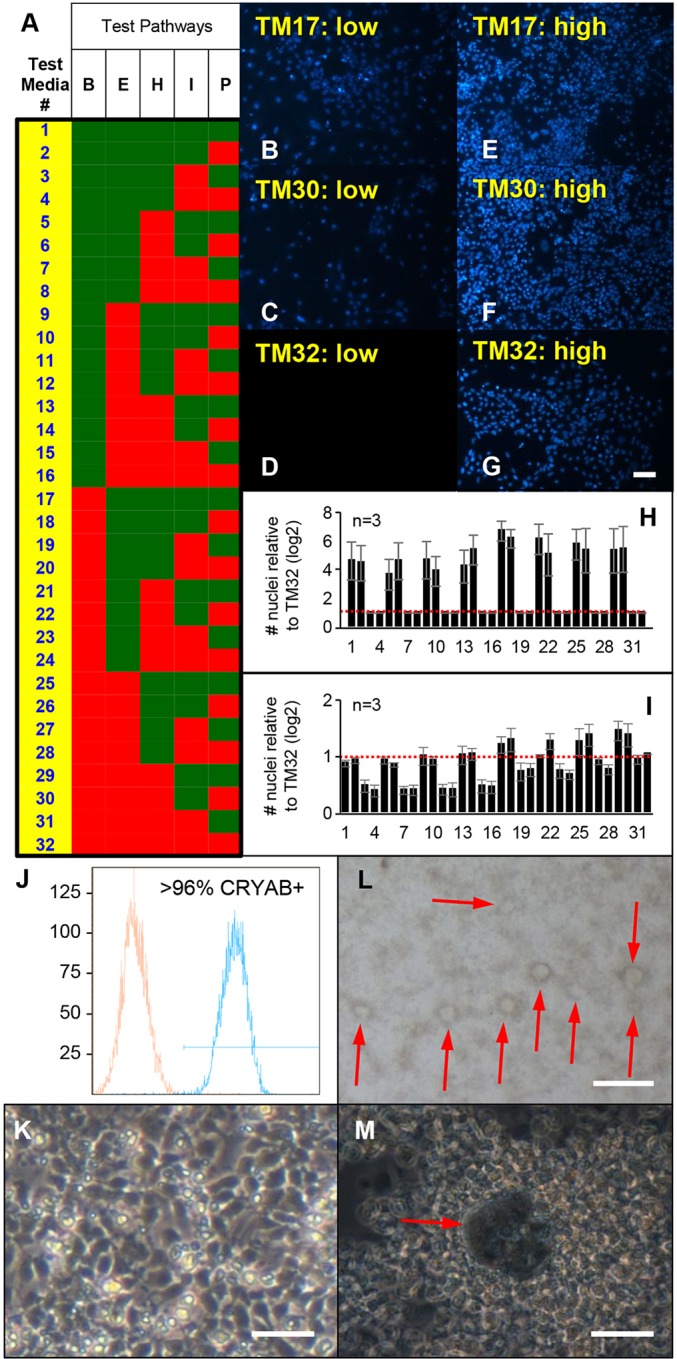
**Combinatorial growth factor screening identified media for ROR1^+^ cell expansion and differentiation.** (A) Schematic diagram showing composition of the test media. FGF2 was included in the basal medium (TM32), with all combinations of the five other test pathways (eight growth factors) tested as shown [B, BMP4, BMP7; E, EGF, TGFα, H, HGF; I, insulin, IGF1; P, PDGF-AA; green square represents factor(s) present; red square represents factor(s) absent]. (B-I) Data from Hoechst-stained ROR1^+^ cells cultured in TM17 (B,E), TM30 (C,F) and TM32 (D,G) after seeding at low (B-D) and high (E-G) cell density, as well as average Hoechst-stained nuclei counts for all media (H,I). These data reveal that TM17 promoted expansion of ROR1^+^ cell cultures while maintaining expression of α- but not β-crystallins (see supplementary material Fig. S3). Scale bar: 20 µm. (J,K) Flow cytometry and light microscopy data show ROR1^+^ cells expanded, frozen, thawed and cultured for 6 days in TM17 retain high levels of CRYAB expression (J) with expected morphology (K) but without detectable expression of β-crystallins (see Table S1 and Fig. S4). Scale bar: 40 µm. (L,M) Light microscopy images show spontaneous production of lentoid-like structures after being expanded, frozen and thawed in TM17, then cultured in stage 2 lens differentiation medium. Cells in these cultures expressed α- and β-crystallins (see Table S2 and Fig. S3). Scale bars: 200 µm in L; 40 µm in M. The data shown in B-I are each representative of three independent differentiation experiments; data are mean±s.e.m. in H,I

To avoid the large vacuolated phenotype seen with initial passaging of the ROR1^+^ cells, a combinatorial growth factor screen was undertaken to test six signalling pathways (nine growth factors) whose receptors are expressed by LECs (Fig. 2A). As FGF signalling is crucial for lens development (Lovicu et al., 2011; Wu et al., 2014) FGF2 was included in the basal medium at 10 ng/ml (TM32; Fig. 2A) – a concentration known to stimulate rat LEC proliferation and migration but not differentiation to fibre cells. All combinations of the remaining five test pathways were assayed on ROR1^+^ cells derived from four hPSC lines. Imaging Hoechst-stained nuclei revealed that media containing insulin and IGF1 (insulin-like growth factor 1) greatly increased ROR1^+^ cell yield (Fig. 2B-I). Mass spectrometry revealed ROR1^+^ cells cultured in these media expressed α- but not β-crystallin proteins (Fig. S3). The high CRYAA sequence coverage obtained indicates that it is one of the most abundant proteins expressed by the cultured ROR1^+^ cells. In contrast, media that contained BMPs had lower cell yield (Fig. 2H,I), and ROR1^+^ cells cultured in these media expressed lens fibre cell crystallin proteins, including CRYBB1, CRYBB2 and CRYBB3 (Fig. S3). As TM17 was among the best-performing proliferation media for ROR1^+^ cells derived from all four PSC lines in both low and high cell-seeding conditions, it was used as the routine ROR1^+^ maintenance medium. TM17 supported ROR1^+^ cell freeze/thawing with retention of α-crystallin protein expression and cell morphology (Fig. 2J,K and Table S1). After ∼2 weeks of high density culture in TM17, or after exposure to media containing FGF2 and BMP4/7, random lentoid production and lens fibre cell crystallin expression was seen (Fig. 2L,M and Table S2).

### Large-scale production of light-focusing human micro-lenses

**Fig. 3.**
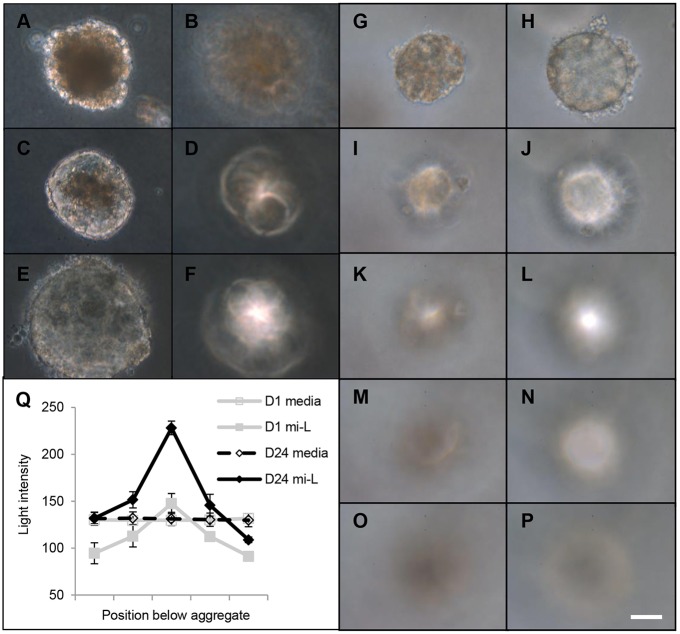
**ROR1^+^ cell aggregation leads to transparent and light-focusing micro-lenses.** (A-Q) Light microscopy data from ROR1^+^-cell aggregates and the maximal focal point below them. After 3 days of culture, the aggregates transmitted less light than the surrounding culture medium (A) and did not focus light (B). As culture progressed, the aggregates transmitted more light (C, day 7; E, day 14) and began focusing light (D, day 7; F, day 14). More-detailed characterisation of a single aggregate shows it had limited transparency (G) and focusing ability (I,K,M,O) on day 3 of culture, but by day 27 it transmitted the same amount of light as the surrounding culture medium (H) and had developed significant focusing ability (J,L,N,P). Quantification of the light transmittance and focusing ability confirms these findings (Q). Scale bar: 40 µm. The images are representative of five micro-lenses from two biological replicates; data in Q are mean±s.e.m.

For controlled and large-scale production of *in vitro* lenses suitable for drug-screening, ROR1^+^ cells underwent forced aggregation to generate small (∼100 µm diameter) LEC aggregates similar to the LEC mass seen during zebrafish lens development. This approach is capable of generating 1200 spherical aggregates per well of a 24-well plate (Fig. S3). These aggregates were embedded in agarose to minimise attachment to each other or the culture dish, and then maintained for up to 6 weeks in stage 3 lens differentiation medium (Yang et al., 2010) on top of the agarose. The cultured aggregates were imaged at various times using phase microscopy (their small size precluding non-phase imaging). Initially, these spherical aggregates transmitted less light than the surrounding culture medium due to an underlying opacity throughout the aggregates (Fig. 3A). However, as culture progressed, this opacity gradually reduced in both size and intensity, such that by ∼2 weeks of culture the aggregates transmitted similar levels of light to the surrounding culture medium (Fig. 3C,E,H,Q). Concomitant with this increase in light transmittance was a striking increase in light-focusing ability. At the beginning of the culture, the cell aggregates displayed very little focusing ability, with the light intensity at the maximal focal point only equivalent to the light intensity of the surrounding culture medium (Fig. 3B). However, as the culture progressed and the light transmittance increased, so too did the light intensity at the focal point below the cell aggregates (Fig. 3D,F). Detailed characterisation of this light-focusing property immediately after aggregation (Fig. 3G,I,K,M,O,Q) compared with after ∼3 weeks of culture (Fig. 3H,J,L,N,P,Q) revealed the cell aggregates developed a remarkable capacity to focus light. In some experiments, some micro-lenses were seen to have clusters of non-transparent cells located adjacent to the micro-lens periphery, likely due to damage during the embedding process (Fig. S4); these clusters tended not to prohibit assessment of light transmittance or focusing ability. Taken together, these data demonstrate that the initial shape and internal composition of the ROR1^+^ cell aggregates is insufficient for either transparency or significant focusing ability, and that gross morphological changes observed over a number of weeks are associated with the development of both maximal light transmittance (relative to the culture medium alone) and maximal light-focusing ability.

### Micro-lens functions are associated with lens fibre crystallin expression

**Fig. 4.**
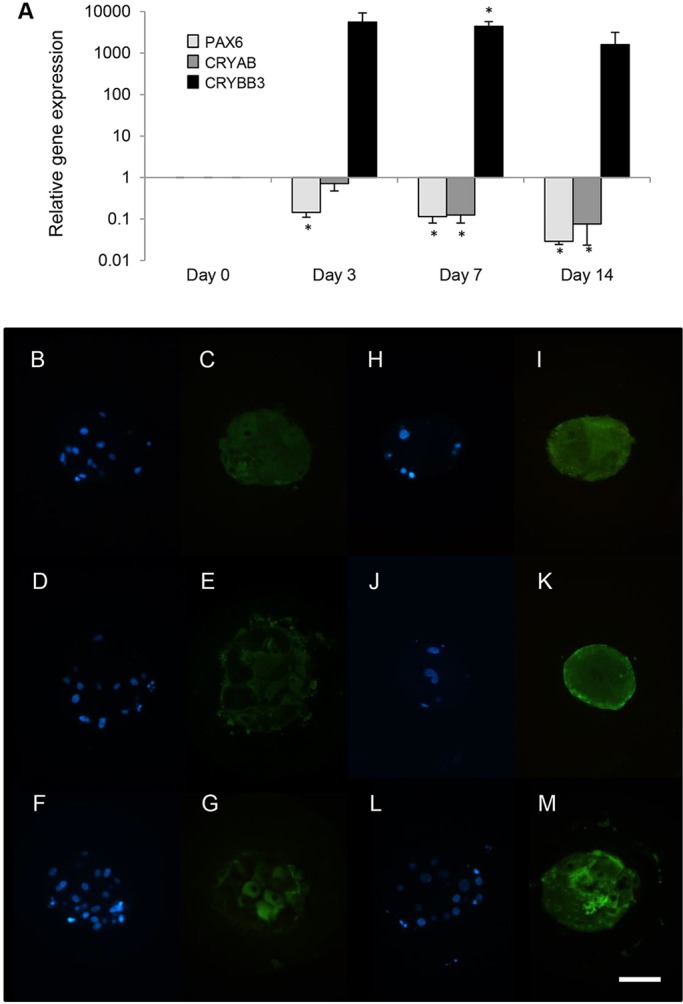
**Aggregation of ROR1^+^ cells induces lens fibre cell crystallin expression.** (A) Real-time PCR analysis of aggregated ROR1^+^ cells results in decreased relative expression of PAX6 and CRYAB, and increased expression of CRYBB3 (*P<0.01; data obtained from four biological replicates and presented as mean±s.e.m.). (B-M) Immunofluorescence analysis shows that after 14 days of culture, αA-crystallin (C) was expressed uniformly throughout the bulk of the micro-lenses, whereas β-crystallin (E) and γ-crystallin (G) were not. After 24 days of culture, αA-crystallin (I), β-crystallin (K) and γ-crystallin (M) were all expressed relatively uniformly throughout the bulk of the micro-lens. The location of DAPI-stained nuclei within the day 14 (B,D,F) and day 24 (H,J,L) aggregates are shown. Scale bar: 40 µm. Each image is representative of five micro-lenses from two biological replicates.

To determine whether the observed changes in light transmittance and focusing ability were associated with changes in crystallin expression, PCR, immunofluorescence and mass spectrometry were used to assess the expression of α-, β- and γ-crystallins at various times during the aggregate culture. The PCR analyses (Fig. 4A) revealed the opposite trend from that seen with ROR1 cell purification (Fig. 1H): i.e. culture of the ROR1^+^ cell aggregates resulted in decreased relative expression of PAX6 and CRYAB mRNA but increased expression of CRYBB3 mRNA. The immunofluorescence analyses showed homogenous expression of CRYAA protein at day 14 (Fig. 4C), whereas expression of both β- and γ-crystallins was sometimes variable (Fig. 4E,G). Immunofluorescence at day 24 showed homogenous expression of CRYAA (Fig. 4I) and more homogenous expression of β- and γ-crystallins (Fig. 4K,M). Expression of lens fibre cell crystallins was similarly supported by mass spectrometry analyses that routinely identified predominantly α- and β-crystallin proteins among the most abundant proteins expressed by the light-focusing micro-lenses (Table S3 and Fig. S5).

### Micro-lens functions are associated with lens fibre cell maturation

**Fig. 5.**
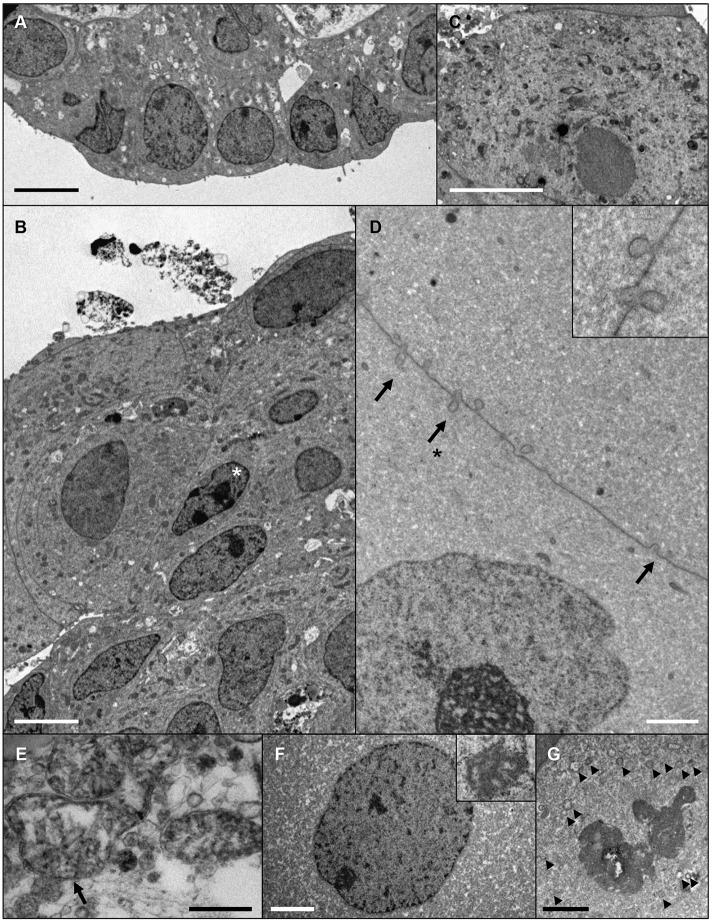
**Evidence of progressive lens fibre cell differentiation in ROR1^+^ cell aggregates.** Electron microscopy data from cultured aggregates. (A,B) A micro-lens cultured for 14 days shows a monolayer of LEC-like cells at the periphery of the tissue (A), and cells with small, rod-shaped nuclei (asterisk) and numerous organelles within the bulk of the tissue (B). (C) LEC-like cell with numerous organelles present at the periphery of a micro-lens after 24 days of culture. (D-G) Ultrastructural indicators of lens fibre cell differentiation within a micro-lens cultured for 42 days. (D) Ball-and-socket type membrane interdigitations (arrows) between adjacent lens fibre-like cells (inset shows a higher magnification of the region indicated with an arrow and asterisk). (E) A swollen mitochondria (arrow). (F) An enlarged nuclei with spoke-like nucleolus (inset). (G) A degraded nuclei with nuclear membrane visible as a series of vesicles (arrowheads). Scale bars: 5 µm in A-C; 2 µm in D,F,G; 0.5 µm in E. Images are representative of seven micro-lenses obtained from two biological replicates.

As specific ultrastructural changes have also been associated with lens fibre cell development, electron microscopy was performed to characterise the cellular organisation within the developing micro-lenses. Early in culture, these analyses revealed LEC-like cells with large nuclei at the periphery of the aggregates (Fig. 5A). At this stage, the bulk of the aggregates consisted of relatively small cells with large nuclei and numerous organelles (Fig. S6A). The nuclei in these bulk cells were typically rod-shaped, with prominent nucleoli and darker nuclear substance compared with the surrounding cytoplasm (Fig. 5B) – similar to the nuclear morphology seen during the early stages of lens fibre cell differentiation *in vivo* (Kuwabara and Imaizumi, 1974; Vrensen et al., 1991).

Later in culture, LEC-like cells could be found at the periphery of the transparent and focusing micro-lenses (Fig. 5C). In larger aggregates (∼200 µm and more diameter) multi-layering of the LECs could be seen that was not apparent in smaller micro-lenses (Fig. S7A). In these later cultures, the bulk of the micro-lenses of all sizes were composed of large cells with varied cross-sectional sizes and relatively homogenous cytoplasm (Fig. S6B,C). These lens fibre-like cells were typically joined by complex membrane interdigitations (Fig. 5D); enlarged and degenerating organelles such as mitochondria could also be found (Fig. 5E) similar to those seen in lens fibre cells *in vivo* (Vrensen et al., 1991). The nuclei within some of these lens fibre-like cells were large and circular-profiled with spoke-like nucleoli (Fig. 5F). In other cells, the nuclear membrane was only recognisable as a chain of vesicles, with some of the nuclear substance appearing to be indistinguishable from the cell cytoplasm (Fig. 5G) – these nuclear morphologies being similar to those seen *in vivo* within terminally differentiating fibre cells of the late bow zone (Kuwabara and Imaizumi, 1974). In some instances, secondary lens fibre-like cells could be seen at the periphery of the aggregates overlying the bulk cells and adjacent to LEC-like cells (Fig. S6D).

### Micro-lenses show evidence of lens capsule formation

As a first step towards investigating production of lens capsule-related material within the ROR1 system, the ROR1^+^ RNA-seq libraries were examined. This analysis revealed ROR1^+^ cells express a range of integrins, collagens and laminins that are known to be required for normal lens development *in vivo* (Table S4). Subsequent immunofluorescence experiments localised laminin and collagen IV expression within the micro-lenses to peripherally located LEC-like cells, which appeared multi-layered in larger micro-lenses (Fig. S8). Electron microscopy revealed the presence of a thin, lens capsule-like material around the micro-lenses that appeared thicker in micro-lenses that had been cultured for longer (Fig. S7A-D).

### Vx-770 induces cataract in micro-lenses

**Fig. 6.**
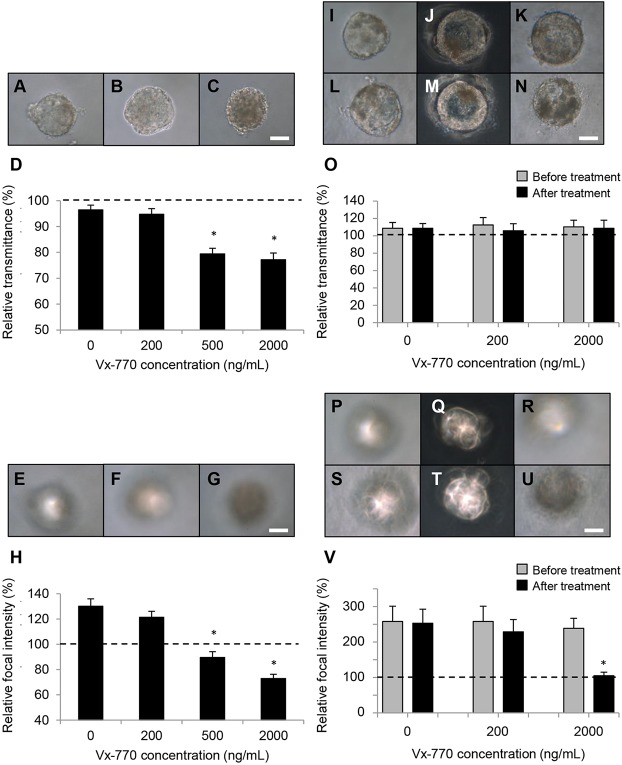
**The CFTR potentiator Vx-770 inhibits light focusing in ROR1 micro-lenses.** (A-D) Light microscopy data showing ROR1^+^ micro-lenses treated with DMSO-only (A) or 200 ng/ml Vx-770 (B) transmitted light at similar levels to the culture medium after 24 days of culture, whereas a micro-lens treated with 2000 ng/ml Vx-770 transmitted less light (C). Scale bar: 40 µm. Quantitative data are shown in D. (E-H) Light microscopy data showing ROR1 micro-lenses treated with DMSO-only (E) or 200 ng/ml Vx-770 (F) had developed focusing ability after 24 days in culture, whereas micro-lenses treated with 2000 ng/ml Vx-770 had not (G). Scale bar: 40 µm. Quantitative data are shown in H. (I-O) Light microscopy data showing micro-lenses treated after they had developed focusing ability. Micro-lenses treated with DMSO-only (I,L), 200 ng/ml Vx-770 (J,M) or 2000 ng/ml Vx-770 (K,N) all transmitted similar levels of light after 7 days of treatment (L-N) compared with before treatment (I-K). Scale bar: 40 µm. Quantitative data are shown in O. (P-V) Light microscopy data showing micro-lenses treated after having developed focusing ability. Micro-lenses treated with DMSO-only (S) and 200 ng/ml Vx-770 (T) retained focusing ability after 7 days of treatment compared with before treatment (P,Q, respectively). A micro-lens treated with 2000 ng/ml Vx-770 focused light prior to treatment (R) but did not after 7 days of treatment (U). Scale bar: 40 µm. Quantitative data are shown in V. In D,O,H,V, *P<1×10^−4^. The data shown (mean±s.e.m.) were each obtained from measurements of 15 micro-lenses from three biological replicates.

The underlying reason for generating functional human lenses *in vitro* was to provide a source of functional human lens tissue for investigating cataract risk factors. To determine whether the micro-lens system might be suitable for investigation of clinically relevant cataracts, the micro-lenses were exposed to Vx-770 – a potentiator of activity for the cystic fibrosis transmembrane conductance regulator (CFTR) protein. This emerging cystic fibrosis drug is reported to have caused cataracts in rats (McColley, 2016). Cataracts have also been reported in children and adolescents receiving Vx-770 (Talamo Guevara and McColley, 2017), and clinical trials are yet to discount an association with its use and cataract formation in paediatric patients (Dryden et al., 2016; McColley, 2016; Van Goor et al., 2009). The concentrations used to test the effect of Vx-770 on ROR1^+^ aggregates (i.e. up to 2000 ng/ml) covered the plasma concentration range reported for children treated with Vx-770 (Davies et al., 2016). When included from the start of culture, the ROR1^+^ cell-aggregates exposed to vehicle or 200 ng/mlVx-770 transmitted similar levels of light compared with the culture medium (Fig. 6A,B). In contrast, the aggregates exposed to 500 ng/ml and higher were less transparent, transmitting significantly less light than the culture medium and the vehicle- and 200 ng/ml-treated aggregates (Fig. 6C,D). Continued culture of these treated aggregates resulted in focusing ability developing in both the vehicle- and 200 ng/ml-treated aggregates (Fig. 6E,F,H), but not the aggregates treated with 500 ng/ml Vx-770 or more (Fig. 6G,H).

To test whether Vx-770 would affect micro-lens function after focusing ability had developed, aggregates were first allowed to develop focusing ability and only then were they exposed to Vx-770. Interestingly, comparison of micro-lens transparency before and after treatment revealed no measurable decrease in light transmittance with any of the treatments (Fig. 6I-O). When comparing micro-lens focusing ability before and after treatment, neither control-treatment nor 200 ng/ml Vx-770 decreased the focusing ability (Fig. 6P,Q,S,T,V). In contrast, micro-lenses treated with 2000 ng/ml Vx-770 showed a large and significant reduction in focusing ability (Fig. 6R,U,V). Notably, these Vx-770-induced effects occurred regardless of the micro-lens size (i.e. from 80 µm to 200 µm in diameter).

## DISCUSSION

### ROR1^+^ cells closely resemble human LECs

The inability to reliably access large amounts of functional human lens tissue has hampered cataract research for decades. Until now, no effective conditions have been identified for simple, robust and large-scale generation of either purified LEC populations or light-focusing lenses, from PSCs of any species. Previous reports of hPSC-based LEC models have been limited by the presence of contaminating non-lens cells, in some cases up to ∼60% non-CRYAA-expressing cells (Yang et al., 2010). Other limitations include the requirement for either: successive rounds of minimally scalable manual purification of LEC progenitor cells that display spontaneous lentoid body formation (Fu et al., 2017; Li et al., 2016); or complex five-colour flow cytometry that requires simultaneous positive and negative selection to obtain small numbers of partially purified LEC progenitors that undergo spontaneous lentoid production (Mengarelli and Barberi, 2013). In contrast, the extensive characterisation data shown here demonstrate simple, robust and large-scale MACS-based purification of ROR1^+^ human LECs. This semi-automated process is capable of generating 10^6^ to 10^8^ ROR1-purified human LECs (from 1×35 mm dish to 6xT175 flasks, respectively). Morphological, transcriptomic and proteomic analyses of these ROR1^+^ cells demonstrate them to be most similar to human LECs, thereby providing a simple and large-scale source of purified human LECs for lens and cataract research.

### ROR1^+^ cells for investigation of posterior capsule opacification

The defined, proliferation-inducing culture conditions identified here provide an extended period of time for investigation of factors that affect human LEC biology compared with previously reported PSC-based lentoid systems. The identification of insulin and IGF1 as significant inducers of ROR1^+^ cell proliferation, and BMPs as inducers of β-crystallin expression, is consistent with known roles for these factors in lens cells from other species (Lovicu et al., 2011). A variety of chick- and rat-based studies have shown that insulin and IGF1 can induce a proliferative response in LECs, and BMP signalling can potentiate aspects of lens fibre cell differentiation (which has also been shown for insulin/IGF1 in some circumstances). The observation that ROR1^+^ cells plated at low density change into large, vacuolated cells with stress fibres suggests they might be suited to investigating posterior capsule opacification (PCO) – the most common complication arising from cataract surgery. The simplicity and scalable nature of the ROR1^+^ cells may provide advantages over existing primary human LEC models of PCO (Wormstone and Eldred, 2016). Ongoing work is aimed at further elucidating how TGFβ signalling integrates with signalling via FGF, insulin and IGF1, BMP and other pathways in ROR1^+^ cells, for comparison with what is currently known of the molecular development of PCO.

### Functional human organoids from non-mammalian developmental templates

Previous work from our group demonstrated that physiologically sized, transparent and light-focusing rat lenses could be generated *in vitro* by mimicking aspects of the lens vesicle stage of mammalian lens development (O'Connor and McAvoy, 2007). These paired explant-derived *in vitro* lenses contained LEC monolayers and bulk compartments of lens fibre cells undergoing terminal differentiation. The desire to generate much smaller yet still functional human micro-lenses (suitable for developmental biology and drug screening applications) led to the hypothesis that partially mimicking aspects of teleost lens development using ROR1^+^ LECs might produce transparent and light-focusing lenses. Zebrafish lens development was chosen as a template as these teleosts have small lenses, and because anatomical features of their lens development have been well described (Greiling and Clark, 2012).

In zebrafish, cells of the surface ectoderm delaminate to form a lens cell mass, rather than a lens vesicle, that measures ∼80 µm in diameter during primary lens fibre cell differentiation. Differentiation of the cells within the lens cell mass forms the primary fibre cells, while at the same time the lens epithelium forms from cells at the periphery of the lens placode. To approximate these events *in vitro*, ROR1^+^ cells underwent forced aggregation to generate spheroidal masses of LECs. The aggregates were then embedded in agarose to minimise attachment to each other or the culture surface, before being asymmetrically exposed to medium containing FGF2 and Wnt3a (Yang et al., 2010) to mimic growth factor delivery to the lens. This approach generated both transparent and light-focusing human micro-lenses, and is the first demonstration of its kind. This approach also provides significantly greater control over the size, timing and location of micro-lens generation compared with existing methods of hPSC-based lentoid generation. Notably, no spontaneous loss of micro-lenses occurs, and the process can generate ∼2.5×10^3^ to ∼2.5×10^5^ light-focusing micro-lenses (from 1×35 mm dish to 6×T175 flasks, respectively). The appearance of LEC multi-layering in the larger micro-lenses (∼200 µm diameter or more, or ∼2.5× the diameter of equivalently staged zebrafish lens masses) suggests there is an upper size limit to the utility of this approach. Nevertheless, the establishment of more anatomically correct functional lens tissue below this size limit (achieved by varying the input cell number during ROR1^+^ cell aggregation) suggests mimicking non-human anatomical templates may be a useful approach for generating small, rudimentary functional tissues for developmental biology and drug screening applications.

### Lens fibre cell differentiation in micro-lenses mimics events seen *in vivo*

The gradual appearance of transparency and light-focusing within the micro-lenses indicates these functional properties developed as a result of specific cellular and molecular changes that occurred within the ROR1^+^ cell aggregates over a period of weeks. Thus, neither the initial shape nor the internal composition of the freshly aggregated ROR1^+^ cells is the main determinant of either transparency or focusing ability.

*In vivo*, the establishment of lens transparency and focusing ability is thought to result from a combination of specific events that occur in parallel. Maintenance of an anterior LEC monolayer occurs while cells at the edges of this monolayer differentiate into primary and then secondary lens fibre cells. At the same time, lens fibre cell production is associated with: cell elongation (the increasing cell size helping to provide the required lens shape); cell alignment (to minimise extracellular light scatter); expression and accumulation of α-, β- and γ-crystallins (to provide the required refractive index); loss of organelles, including the nucleus (to remove intracellular light-scattering particles); and accumulation of complex membrane interdigitations (to maintain maximal alignment of the fibre cells and thus minimise the possibility of light-scattering due to intercellular spaces).

Loss of the opaque phenotype initially seen in the aggregated ROR1^+^ cells, and concomitant development of transparency and light focusing, appear to recapitulate key aspects of the above lens-development processes. Regions of LEC-like cells were present at the periphery of the aggregates. Within the bulk of the aggregates, lens fibre-like cells became larger and their cross-sectional profiles were varied, as seen in mouse, bovine and chick primary lens fibre cells (al-Ghoul and Costello, 1997; Shestopalov and Bassnett, 2000; Taylor et al., 1996). The cytoplasm of these fibre-like cells became more homogenous as β- and γ-crystallins were expressed, with some evidence suggestive of rudimentary secondary fibre cell production. Organelles in these lens fibre-like cells showed evidence of being degraded, including progressive appearance of classic nuclear degradation morphologies indicative of terminal lens fibre cell denucleation (i.e. rod-shaped nuclei early in culture; later in culture spoke-like nucleoli and evidence of nuclear membrane breakdown) (Kuwabara and Imaizumi, 1974; Vrensen et al., 1991). The lens fibre-like cells also accumulated complex membrane interdigitations that are characteristic of the lens, such as ‘ball-and-socket’ type junctions, a feature not described before in other PSC-derived lentoids.

Production of the lens capsule and expression of associated integrins are also crucial elements of lens development; loss of capsule components or related integrins leads to lens malformations (Walker and Menko, 2009; Wederell and de Iongh, 2006). The data presented here shows ROR1^+^ LECs express transcripts for key extracellular matrix molecules and lens-related integrins. Additionally, LEC-like cells within the light-focusing micro-lenses expressed laminin and collagen IV. Thin, nascent lens capsule-like material could also be found, of a similar thickness to that seen with published stem cell-derived lentoids (Fu et al., 2017; Li et al., 2016; Yang et al., 2010). These observations – when combined with evidence of LEC maintenance, terminal lens fibre cell differentiation and well-contained lens substance in the majority of micro-lenses – suggest that integrin-related signalling pathways are being sufficiently stimulated for development and maintenance of transparency and focusing. The role of this lens capsule-like material in development of the ROR1^+^ micro-lenses could be further tested, e.g. by generating micro-lenses from hPSCs that have been derived from individuals known to develop lens capsule-related cataract, such as those with Alport syndrome (Chen et al., 2015; Song et al., 2011).

### Towards a systems biology blueprint for development of lens function

Given that key features of normal lens development were observed as the micro-lenses become transparent and focused light, these micro-lenses may be useful for defining and testing an emerging systems biology blueprint for development of lens function. Namely, how signalling via defined growth factors leads to integration of multiple intracellular signalling cascades that activate progressive transcriptional changes (and other changes – membrane dynamics, protein packing, etc.) that lead to establishment of a functional three-dimensional tissue. Decades of important research have identified growth factors (including FGFs, insulin and IGF, BMPs, Wnt) and related kinases (Lovicu et al., 2011), as well as transcription factors (PAX6, FOXE3, etc.) and target genes (Cvekl and Zhang, 2017) required for normal lens development – and how some of these elements interact. Recent studies have added to this body of knowledge, with this new information yet to be fully incorporated with prior knowledge (Anand and Lachke, 2017). The defined ROR1^+^ cell type and associated culture media of the micro-lens system suggests it may be possible to integrate this new information to create and test a comprehensive molecular blueprint for development of lens transparency and focusing, e.g. via growth factor variation, time-course transcriptional profiling, as well as gain- and/or loss-of-function studies. Interesting studies could include investigation of lens capsule production, which appears less extensive in all of the PSC-based lentoid systems compared with the normal lens. Similarly, variability in the timing of denucleation between fibre-like cells has been described in each of the PSC-based lentoid systems. These observations could be due to influences such as the limited growth factor set used to induce lens fibre cell differentiation (i.e. FGF2 and Wnt3a), and/or the relatively large space around the lentoids (compared with the lens *in vivo*) that could alter the concentration, and therefore effectiveness, of autocrine and paracrine factors. The ROR1^+^ micro-lens system has the potential to further interrogate these issues and provide human-specific information related to development of lens function. A human-specific lens development blueprint could also have relevance for understanding how changes resulting from congenital mutation, environmental insults and ageing lead in isolation or combination to presbyopia and cataracts.

### Investigating human cataracts using ROR1^+^ cells and micro-lenses

The clinical relevance of the micro-lens system is supported by the finding that focusing ability was decreased by treatment with high concentrations of Vx-770 (at the upper-range of minimum circulating plasma concentrations detected in paediatric patients treated with Vx-770). This loss of focusing ability occurred regardless of the timing of Vx-770 treatment (i.e. with treatment before or after development of micro-lens focusing) and independently of micro-lens diameter.

Nuclear and posterior subcapsular cataracts have been noted in rat pups treated with Vx-770, and cataracts have also been noted in child and adolescent cystic fibrosis patients treated with Vx-770. Eye examinations are therefore recommended for children being treated with Vx-770 (Talamo Guevara and McColley, 2017). Little-to-no detail is available on the histology or mechanism of Vx-770-induced cataract in either rats or humans. Thus, the ROR1^+^ micro-lens system appears to be a relevant and useful human model of lens function for future investigations into the molecular mechanisms of Vx-770-induced cataract (e.g. assessing protein aggregation, changes to membrane properties, vacuolisation, cell death, etc.). The findings that neither transparency nor focusing ability developed when Vx-770 was included from the start of culture suggests that LECs are affected by high Vx-770 concentrations. Whether the lens fibre-like cells are also affected needs to be determined – a possibility based on the loss of focusing observed when Vx-770 treatment was applied after focusing had developed.

The Vx-770 data also suggest that the micro-lens system may be a relevant model for investigating other known cataract risk factors, the mechanisms of action of which are yet to be fully defined (e.g. genetics, age, diabetes, smoking, UV light, radiation, drugs, etc.) (Robman and Taylor, 2005; Shiels and Hejtmancik, 2017). Recent studies have identified small molecules that can reverse some forms of cataract that arise due to protein aggregation (Makley et al., 2015; Quinlan, 2015; Zhao et al., 2015), though their efficacy in individuals with cataracts remains to be demonstrated. Although protein aggregates have been identified in cataractous human lenses, the common and unique molecular events initiated by different cataract risk factors are currently unclear. Moreover, other particles that appear distinct from protein aggregates have been identified in human lenses that may account for the light-scattering associated with some forms of cataract (Costello et al., 2010; Gilliland et al., 2001). Thus the micro-lens system holds potential for elucidating cataract mechanisms resulting from individual risk factors, and for identifying additional candidate anti-cataract therapeutic targets.

### Summary

The cellular, molecular and functional features of human ROR1^+^ LECs and micro-lenses suggest they share sufficient similarities with human LECs and lenses to provide a useful *in vitro* tool with which to investigate lens and cataract development. In addition, the simplicity, scalability and defined nature of these systems represent a significant advance over existing hPSC-based approaches. The ROR1^+^ LECs and micro-lenses will enable: functional genomic studies with relevance to developmental biology; investigation of PCO and a wide variety of primary cataract risk factors; clinical toxicity assays; as well as targeted and/or high-throughput anti-cataract drug screening. The capacity to generate disease-specific hPSCs suggests the micro-lenses are also a likely platform for investigating a wide range of poorly understood whole-body syndromes that include cataract as a symptom. For all of these studies the micro-lenses provide a large-scale, predictable, robust and highly purified human system with two reliable and fundamentally appropriate functional assays: the ability to quantify effects on lens transparency and on focusing ability.

## MATERIALS AND METHODS

### Pluripotent cell culture

Human pluripotent cells were obtained as follows: embryonic stem cells were provided by A. Nagy (CA1 line) (International Stem Cell Initiative et al., 2007) and the StemCore facility (MEL1 line), University of Queensland, Australia; induced pluripotent stem cells hiPSC-TT and hiPSC-LacZ were obtained from E. Stanley and A. Elefanty, Murdoch Children's Research Institute (Melbourne, Australia). Approval for use of these cells was obtained from the Western Sydney University Human Research Ethics Committee (Australia). Pluripotent cells were cultured in mTeSR1 (StemCell Technologies) on plates coated with Matrigel (Corning), and passaged as clumps using 1 mg/ml dispase as previously described (O'Connor et al., 2008a). For differentiation experiments, pluripotent cells were plated as single cells on Matrigel-coated dishes and cultured in mTeSR1 until confluent, after which the cells were exposed to the stage 1 lens differentiation medium.

### Lens differentiation and ROR1^+^ cell separation/culture

A three-stage differentiation protocol was used to generate heterogeneous cultures containing lens cells (Yang et al., 2010). Growth factors were sourced from Miltenyi Biotec and Peprotech, and the base medium for each stage was DMEM:F12 (Thermo Fisher Scientific). Initial modification of this protocol involved increasing the concentration of noggin to 500 ng/ml and including 10 nM SB431542 in the stage 1 medium, followed by reducing the concentration of FGF2 to 10 ng/ml in stage 3. For purification of ROR1^+^ cells via magnetic cell separation, single-cell suspensions were obtained using TrypLE (Thermo Fisher Scientific) during stage 2 of the lens differentiation protocol. The cells were then incubated with a biotinylated anti-human ROR1 antibody (BioScientific; AF2000) and labelled cells purified using anti-biotin microbeads and an autoMACS cell separator (Miltenyi Biotec). Purified ROR1^+^ cells were plated on Matrigel-coated dishes in M199 medium (Thermo Fisher Scientific) containing 10 ng/ml of FGF2 or in test media (TM) consisting of M199 and combinations of the following growth factors: BMP4/BMP7 (20 ng/ml each); EGF/TGFα (5 ng/ml each); HGF (10 ng/ml); IGF1/insulin (10 ng/ml and 10 µg/ml); and PDGF-AA (10 ng/ml). All four human pluripotent stem cell lines (two embryonic and two induced pluripotent) tested behaved similarly.

### Micro-lens formation, culture and focal point image analysis

ROR1^+^ cells were aggregated via centrifugation at 300 ***g*** for 5 min using AggreWell plates (StemCell Technologies; 1200 micro-wells per 24-well plate well). Aggregates were cultured in the plates for 1 to 2 days before being collected and embedded in 0.25% agarose (Amresco) in M199, and then cultured for up to 42 days in stage 3 medium described above. At various times during culture, the developing micro-lenses were assessed for light transmission and focusing ability via light microscopy, using an adaptation of our published method for assessment of *in vitro*-regenerated rat lenses (O'Connor and McAvoy, 2007; O'Connor et al., 2008b). Briefly, using an inverted microscope, individual micro-lenses were brought into focus and an image taken. The objective lens was then lowered until a focal point was reached, at which location another image was taken and the distance travelled to this point recorded. The objective lens was lowered again an equivalent distance and a third image taken. The objective lens was then raised and two more images taken, half-way between the first and second images and the other half-way between the second and third images. Measures of transmitted light and focal points were obtained by quantifying the grey-level approximately within the central quarter diameter of each micro-lens using ImageJ. Measurements were compared using the Student's *t*-test and are shown as mean±s.e.m.

### Flow cytometry

Single cell suspensions of differentiated cells were obtained using TrypLE and stained as previously described (O'Connor et al., 2008a,b; Ungrin et al., 2007). Primary antibodies used included anti-human ROR1 (BioScientific) and anti-human CRYAB (ENZO Life Sciences; ADI-SPA-223); the secondary antibody used was an AlexaFluor-488 anti-IgG antibody (Thermo Fisher Scientific; A11001). Labelled cells were analysed using a MACSquant cell analyser (Miltenyi Biotec) and data analysed using the Student's *t*-test (mean±s.e.m.).

### RNA-seq, PCR and *in situ* hybridisation

RNA from ROR1^+^ cells was collected immediately after cell separation and purified using an Isolate II RNA purification kit (Bioline) as per the manufacturer's instructions. The quality of purified RNA samples was assessed using a Bioanalyser (Agilent) before RNA-seq libraries were prepared using a TruSeq Stranded Total RNA Sample Preparation Kit (Illumina). Samples were sequenced on an Illumina HiSeq 2500 instrument using 2×100 paired end reads, and the data have been deposited in GEO (http://www.ncbi.nlm.nih.gov/geo/) under accession number GSE94296. Reads were analysed using FastQC to assess the quality of the data and processed using the Falco framework (Yang et al., 2016), with HISAT2 (Kim et al., 2015) as the aligner and featureCounts (Liao et al., 2014) as the read quantification tool (extra arguments for featureCounts: -s 2 -t exon -g gene_name --primary). The hg19 genome reference and GTF annotation used for building the alignment index and quantification, respectively, were obtained from GENCODE (GRCh37.p13) (Harrow et al., 2012). Gene expression was normalised as reads per million reads (RPM) per sample. For principal component analysis of cell-type specific genes, the ROR1^+^ RNA-seq data was compared against a set of published gene expression data that consisted of adult human LECs and lens fibre cells (Hawse et al., 2005), as well as various ENCODE samples, including foetal lens tissue, pluripotent stem cells, and other foetal and adult tissues. To minimise the batch effect when comparing gene expression profiles from different data sets, expression values were discretised to a value of 1 for the 1000 most highly expressed genes in each cell type, or 0 otherwise. Genes expressed in >20% of the datasets were removed before computing a pairwise dissimilarity matrix using the binary distance function in R. The dissimilarity matrix was then used as the input to the principal component analysis (PCoA) function implemented in an R package CIDR (Lin et al., 2017). Results were visualised using the first two principal components (PC1 and PC2). For gene-set analysis against published gene expression data, a large compendium of tissue-specific gene expression was generated consisting of 144 human and 94 mouse ENCODE datasets, as well as published lens transcriptomic data sets for human (Hawse et al., 2005) and mouse (Hoang et al., 2014; Khan et al., 2015; Lachke et al., 2012) (i.e. 145 human and 95 mouse cell/tissue types total). Where more than one replicate was available for any tissue or cell type, the mean expression value for each gene was calculated. To ensure a consistent gene universe across this large compendium, non-ubiquitously mapped gene symbols were removed. To generate a compendium of cell/tissue-specific marker genes, the top 3000 highest expressed genes in each tissue were filtered to only keep genes that were highly expressed in 5% or fewer tissue/cell types. The highest-expressed ROR1^+^ cell-specific genes were determined using the same approach, and then compared against the compendium of cell/tissue-specific marker gene set using Fisher's exact tests and Benjamini-Hochberg correction on the resulting *P*-values (Djordjevic et al., 2016). For semi-quantitative real-time PCR analysis, cDNA was synthesised from >100 ng purified RNA using Bioscript (Bioline) and a Mastercycler (Stratagene). Semi-quantitative real-time PCR was performed using Go-Flexi Taq and SYBR Green (Bioline) and an MX3005P real-time PCR machine (Agilent Technologies); PCR primer sequences used are shown in Extended Data Fig. 1. Data were analysed using Student's *t*-test (mean±s.e.m.). *In situ* hybridisation analysis was performed using the mouse embryo *in situ* hybridisation resource at www.genepaint.org.

### Teratoma assay

All experiments involving animals were approved by the Animal Research Ethics Committee at Western Sydney University. Assessment of the teratoma-forming ability of purified, ROR1^+^ cells was undertaken as previously described (O'Connor et al., 2011). Grafts containing single cell suspensions of 10^6^ cultured ROR1^+^ cells were transplanted in 100 µl of ∼10 mg/ml Matrigel under the back flank of 12-week-old NOD/SCID mice; grafts were randomly assigned amongst littermates, with the number of mice used minimised by transplanting multiple grafts in each animal. Control grafts contained, in addition to the ROR1^+^ cells, single-cell suspensions of up to 5×10^5^ undifferentiated pluripotent cells. Mice were housed for up to 12 weeks post-transplantation, euthanized with CO_2_, and grafts were fixed in 10% neutral buffered formalin and assessed without blinding.

### Immunofluorescence staining

Cultured micro-lenses were fixed at room temperature without removal from the surrounding agarose which was ∼2 mm thick. Fixation was performed with 10% neutral buffered formalin for 1 h for laminin and collagen IV detection (though section shrinkage was noted) or 24 h for crystallin detection (leakage of crystallins into the surrounding agarose was noted at lower fixation times). The fixed agarose samples containing micro-lenses were washed three times with phosphate-buffered saline. Samples were dehydrated in a Microm STP-120 Tissue Processor (Thermo Fisher Scientific) in 50%, 70% and 80% ethanol (each for 60 min), followed by 90% and 100% ethanol (each 2×90 min), xylene (3×90 min) and paraffin at 60°C (1×60 min and 1×90). Samples were embedded in paraffin and 5 µm sections cut. Immunofluorescent staining was performed as previously described (O'Connor and McAvoy, 2007; [Bibr DEV155838C30][Bibr DEV155838C31]) using the following anti-human primary antibodies at ∼4 µg/ml (Santa Cruz Biotechnology): anti-CRYAA (sc-22743); anti-β-crystallin (sc-22745); and anti-γ-crystallin (sc-22746). Control primary antibody staining was performed using rabbit IgG (Innovative Research; 121266101). Secondary antibody staining was performed using an Alexafluor-488 anti-rabbit IgG antibody (Thermo Fisher Scientific; A11078). Nuclei were counterstained with 1 µg/ml Hoechst or DAPI (Thermo Fisher Scientific). Images were photographed using a CKX-41 microscope (Olympus) and digital camera with QCapture 6 software (QImaging); images are shown with no digital manipulation.

### Mass spectrometry

For detection of only the most-abundant proteins, cultured ROR1^+^ cells or whole micro-lenses were collected directly from culture in 15 µl of 0.5% RapiGest SF (Waters) in 50 mM NH_4_HCO_3_. Samples were homogenised on ice for 5 min before reduction with 100 µl of 5 mM dithiothreitol (Cabriochem) in 50 mM NH_4_HCO_3_ for 1 h at 60°C, then alkylated with 100 µl of 15 mM iodoacetamide (Merck) in 50 mM NH_4_HCO_3_ for 1 h at room temperature. Samples were proteolysed overnight at 37°C with 10 ng/ml of trypsin (Promega) in 75 mM NH_4_HCO_3_. Peptides were purified by solid phase extraction using Waters Oasis HLB cartridges (30 mg, 1 ml). Pre-cleaning with 1 ml acetonitrile (ACN) was followed by conditioning with 1 ml 0.1% trifluoroacetic acid (TFA). Samples were acidified with 250 μl of 0.4% aqueous TFA prior to loading. Samples were washed consecutively with 1 ml of 0.1% TFA to remove salts, 1 ml of ultrapure H_2_O to remove aqueous soluble contaminants and TFA, then peptides were eluted into low-binding microcentrifuge tubes using 500 μl of 70% aqueous ACN. Solvents were removed using rotational vacuum concentrator for 2-3 h. Dried peptide samples were treated with 15 μl of 0.1% aqueous formic acid and rested for 30 min. Samples were triturated prior to centrifugation at 14,000 ***g*** for 10 min. Supernatants containing peptides were analysed by LC-MS/MS using a nanoAcquity UPLC and Xevo QToF mass spectrometer (Waters); 3 μl of sample were loaded at 3 μl/min onto a C18 Symmetry trapping column of dimensions 180 µm×0 mm (Waters) and desalted at this flow rate for 5 min using 1% ACN in water with 0.1% formic acid. Peptides were washed off the trap at 400 nl/min onto a C18 BEH analytical column (Waters) packed with 1.7 μm particles of pore size 13 nm of dimensions 100 µm×100 mm, using a ramped method from 1% to 85% ACN (with 0.1% formic acid) over 37 min. Eluting peptides were identified by MS/MS using a Xevo QToF mass spectrometer (Waters), fitted with a nanospray source with an emitter tip tapered to 10 µm at 2300 V in positive ion mode. Data-dependent acquisition was performed with continuous scanning for 2^+^ to 4^+^ charged peptides, an intensity of >50 counts and a maximum of three ions in any given 3 s scan (precursor peptides were excluded for 30 s). The MS/MS data files were analysed using Mascot Daemon and queried against the SwissProt database using Homo sapiens-specific searches. Variable modifications of carbamidomethyl (C), deamidated (NQ), oxidation (M) and propionamide (C) were used with peptide and MS/MS mass tolerances of 0.05 Da. Only peptide hits with *P*<0.05 were reported. Peptides identified by Mascot were further validated by manual inspection of the MS/MS spectra for the peptide to ensure the b- and y-ion series are sufficiently extensive for an accurate identification. Percolator-based decoy searches (Käll et al., 2007) were also performed on the samples, and these revealed false discovery rates of 0%.

### Electron microscopy

Micro-lenses were fixed for 1 h at room temperature in 2.5% glutaraldehyde in 0.1 M phosphate buffer, pH 7.4. A 3 mm biopsy punch and tweezers were used to isolate micro-lenses from the agarose gel. Samples were then fixed in 2.5% glutaraldehyde for a further 48-72 h at 4°C, after which they were washed four times at hourly intervals with 5 ml phosphate buffer at 4°C. Samples were then transferred to 0.1 M sodium cacodylate buffer (pH 7.4) for 2 h, post-fixed in 2% OsO_4_ for 4 h then rinsed in cacodylate buffer. Samples were then stained with 1% tannic acid for 30 min at room temp and rinsed in cacodylate buffer. This was followed by rinsing in 2% sodium acetate, en bloc staining with 2% uranyl acetate for 1 h, dehydration in a series of graded alcohols and dry acetone, infiltration with Spurr's resin diluted in acetone, and polymerisation in 100% standard hardness Spurr's resin at 70°C. The embedded micro-lenses were sectioned at 90 nm using a Powertome ultramicrotome (RMC Boeckeler) and imaged with a Morgagni 268D transmission electron microscope (FEI) at 80 kV.

## Supplementary Material

Supplementary information

Supplementary information
