# Supplementary material for: Light-focusing human micro-lenses generated from pluripotent stem cells model lens development and drug-induced cataract in vitro
Source: Development. 2018 Jan 1;145(1):dev155838. doi: 10.1242/dev.155838 (PMC5825866; doi:10.1242/dev.155838)
Supplement: Supplementary information [file develop-145-155838-s1.pdf]

Supplementary material Fig. S1

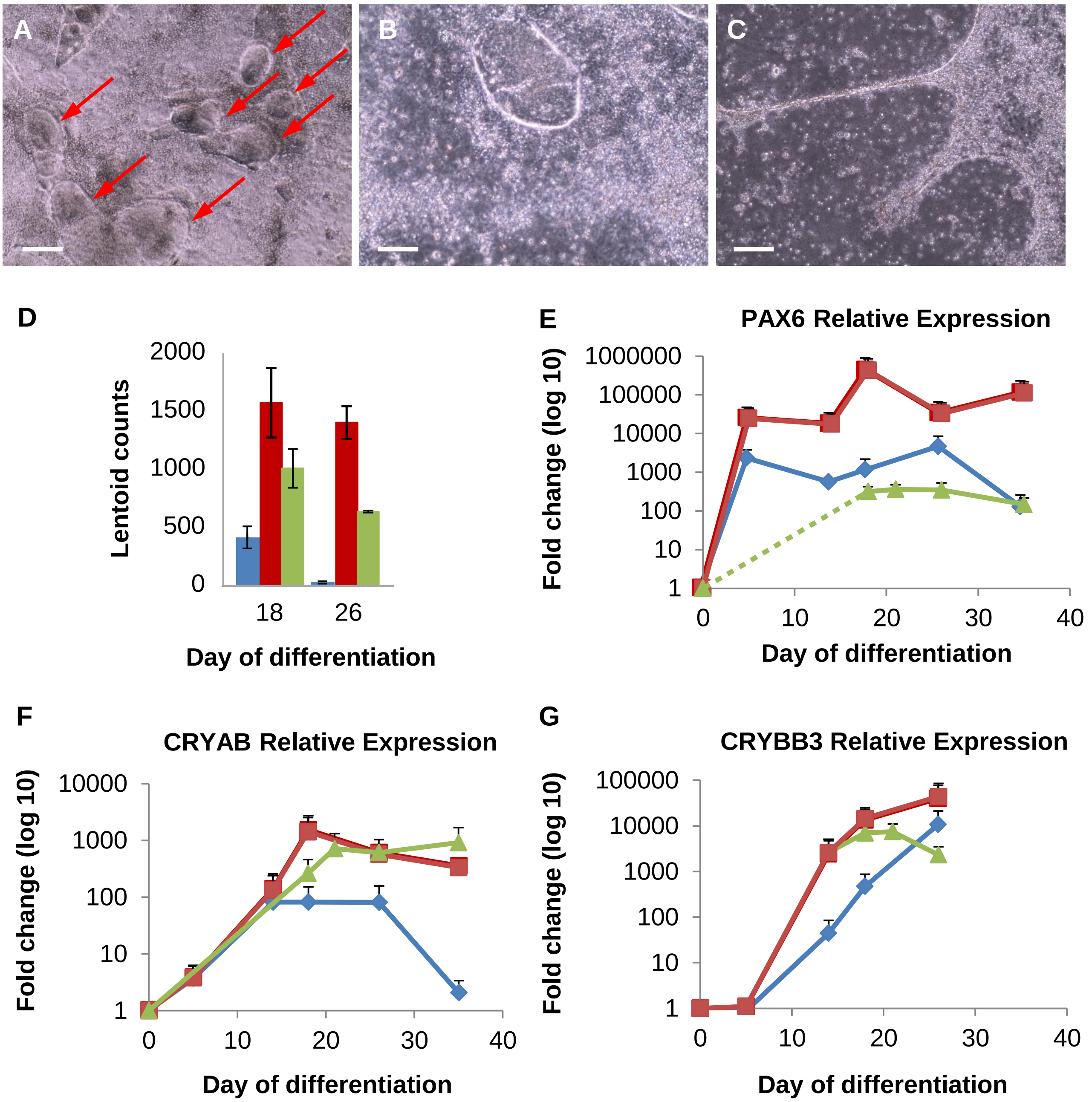

H

**Primer**	**Forward**	**Reverse**
GAPDH	CCCATCACCATCTTCCAGGAG	CTTCTCCATGGTGGTGAAGACG
PAX6	CCCCACATATGCAGACACAC	TCACTTCCGGGAACTTGAAC
CRYAB	TGATTGAGGTGCATGGAAAA	ATCAGCTGGGATCCGGTATT
CRYBB3	CCGCAAGATGGAGATAGTGG	ACTCATAGCCAACCCACGTC

Development • Supplementary information

Supplementary Figure S1. Modification of the 3-stage lens differentiation protocol increased lentoids and lens gene expression but did not produce pure LEC populations. (A-C) Images of lentoids (red arrows) generated after modification of the published 3-stage lens differentiation protocol (Yang et al., 2010). Increasing the concentration of Noggin from 100 to 500 ng/mL and including 10 nM SB431542 (Activin/BMP/TGF-β pathway inhibitor) in the Stage 1 medium produced large numbers of lentoids (A). Some lentoids had light-refractive borders (B), however they did not focus light. As described in the published protocol, the lentoids detached from the culture surface and were lost when changing the culture medium (C). Scale bars, 500 μm, 100 μm and 500 μm, respectively. (D-G) Time-course comparison of lentoid production and lens-related gene expression between the published 3-stage lens differentiation protocol (blue) and two modified protocols (red, green; n = 3). Increasing the concentration of Noggin to 500 ng/mL and including 10 nM SB431542 in the Stage 1 medium (red) increased: the number of lentoids produced per 35mm dish and the time they were retained in the culture (D); PAX6, CRYAB and CRYBB3 expression as detected by semi-quantitative real-time PCR (E-G). Subsequently reducing the concentration of FGF2 in Stage 3 from 100 ng/mL to 10 ng/mL (green) in an attempt to maintain LECs decreased lentoid production (D) as well as PAX6 and CRYBB3 mRNA expression, while maintaining CRYAB expression (E-G: green). Despite this, heterogeneous morphologies and random lentoid production still occurred (A-C). (H) PCR primers used for analysis of differentiating cells. The data shown in A-G were obtained from 3 independent differentiation experiments.

Development • Supplementary information

Supplementary material Fig. S2

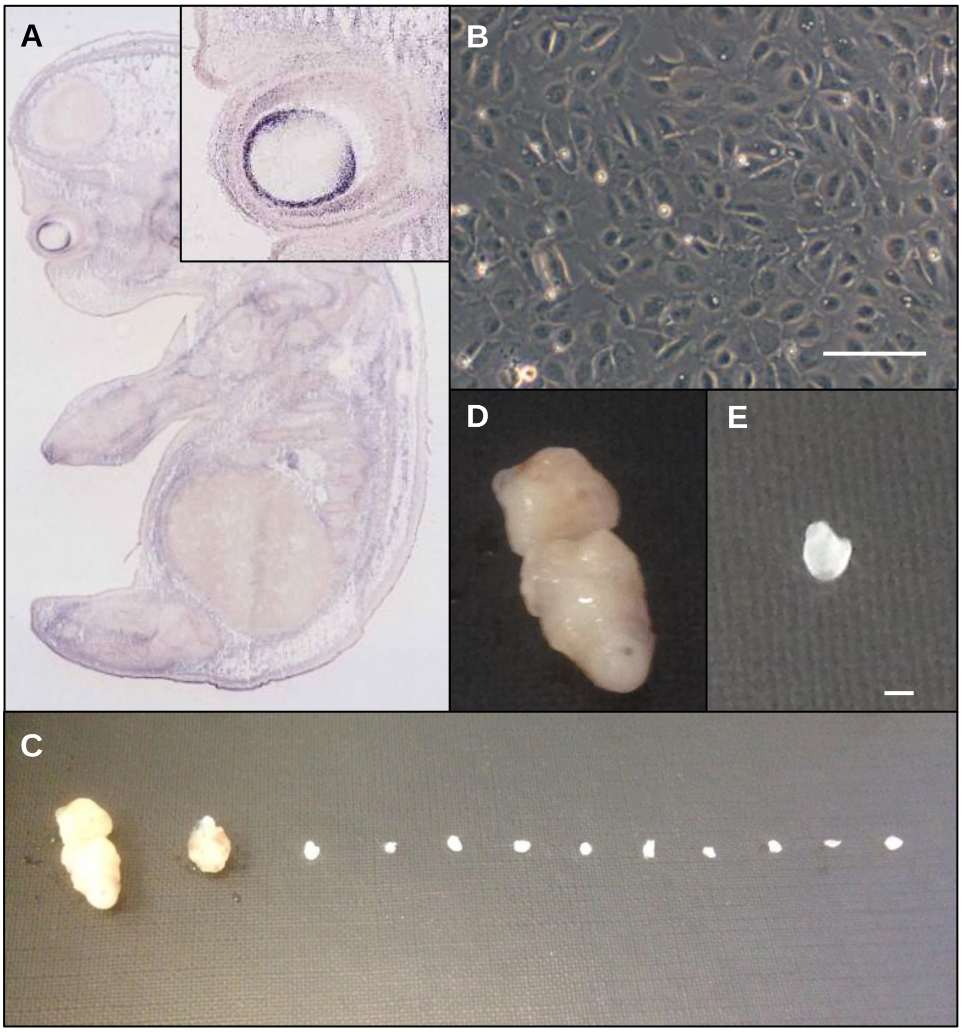

Supplementary Figure S2. ROR1 expression in embryos and ROR1 teratoma assay data. (A) In situ hybridisation data (www.genepaint.org) showing ROR1 transcript expression is predominantly expressed by LECs at embryonic day 14. (B) ROR1+ cells plated at high cell densities after purification showed uniform polygonal morphologies (cells shown 2 days after plating). Scale bar: 100 µm. (C) Twelve grafts, each containing 10^6 ROR1+ cells, show that teratoma formation only occurred when undifferentiated pluripotent cells were deliberately included with the ROR1+ cells in control grafts (i.e., the 1st and 2nd grafts on the left were seeded with 500,000 and 50,000 disaggregated pluripotent stem cells, respectively, equivalent to ~5,000 and 500 colony-forming cells). (D) Higher magnification of the left-hand control graft shown in (C). (E) Higher magnification of a graft that received only ROR1+ cells. Control grafts were collected 6 weeks after transplantation; all other grafts were collected 12 weeks after transplantation. Scale bar: 1 mm.

Development • Supplementary information

Supplementary material Fig. S3

A

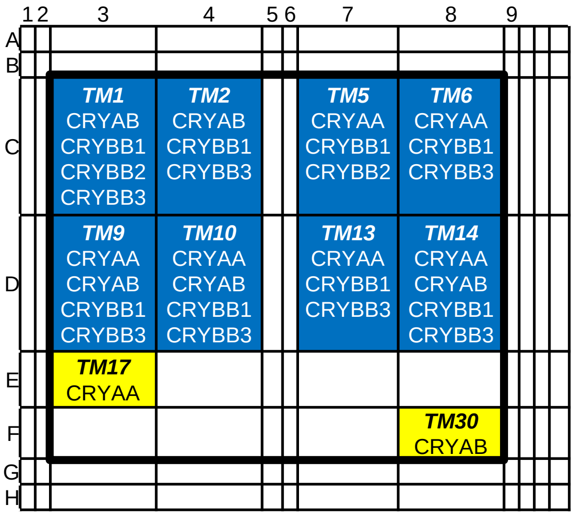

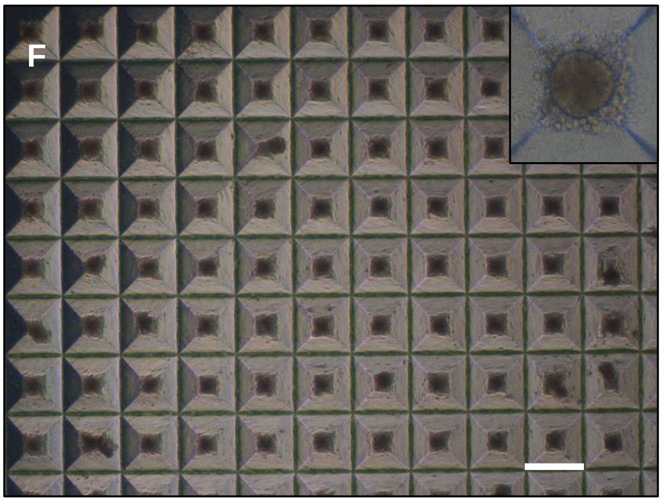

B

1 MDVTIQHPWF KRTLGPFYPS RLFDQFFGEG LFEYDLLPFL SSTISPYYRQ
51 SLFR**TVLDSG ISEVR**SDRDK FVIFLDVK**HF SPEDLTVK**VQ DDFVEIHGKH
101 NERQDDHGYI SREFHRRYRL PSNVDQSALS CSLSADGMLT FCGPK**IQTGL**
151 **DATHAERAIP VSREEKPTSA PSS**

C

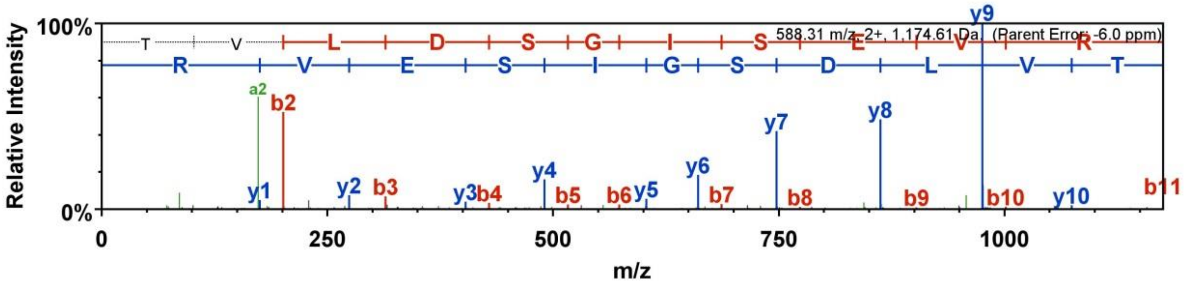

D

1 MSQAAK**ASAS ATVAVNPGPD TK**GKGAPPAG TSPSPGTTLA PTTVPITSAK
51 AAELPPGNYR LVVFELENFQ GRRAEFSGEC SNLADRGFDR VRSIIVSAGP
101 WVAFEQSNFR GEMFILEKGE YPRWNTWSSS YRSDRLMSFR PIKMDAQEHK
151 ISLFEGANFK GNTIEIQGDD APSLWVYGFS DRVGSVK**VSS GTWVGYQYPG**
201 **YR**GYQYLLEP GDFRHWNEWG AFQPQMQSLR RLRDKQWHLE GSFPVLATEP
251 PK

E

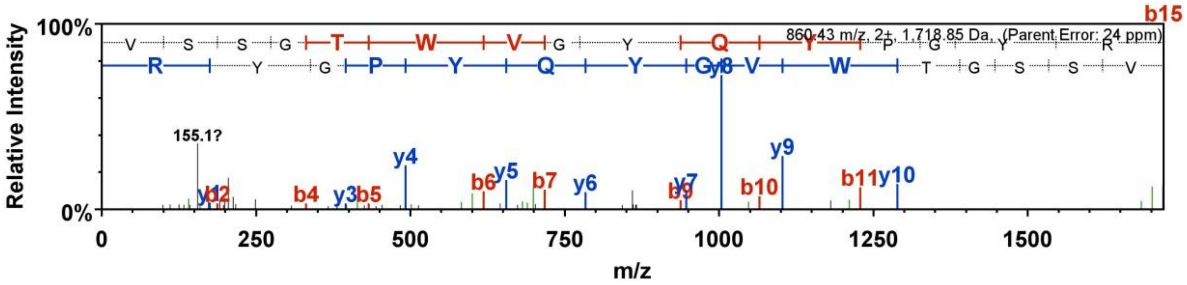

Development • Supplementary information

Supplementary Figure S3. Mass spectrometry analysis of ROR1+ cells cultured in TM17 express LEC but not lens fibre cell crystallins. (A) Schematic diagram summarising the crystallin proteins detected in ROR1+ cells cultured in the test media. (B) MS/MS analysis revealed 28% CRYAA protein sequence coverage obtained from ROR1+ cells expanded, frozen, thawed and then cultured for 6 days all in TM17. (C) Raw mass spectrometry data showing identification of the CRYAA peptide underlined in (B). (D) MS/MS analysis revealed 12% CRYBB1 protein sequence coverage obtained from ROR1+ cells expanded, frozen and thawed in TM17 and then cultured for 6 days in Stage 2 lens differentiation medium. (E) Raw mass spectrometry data showing identification of the CRYBB1 peptide underlined in (D). These data are representative of data obtained from 3 independent differentiation experiments. (F) Light microscopy image showing relatively homogenous and large-scale production of ROR1+ cell aggregates. Scale bar: 400 μm.

Development • Supplementary information

Supplementary material Fig. S4

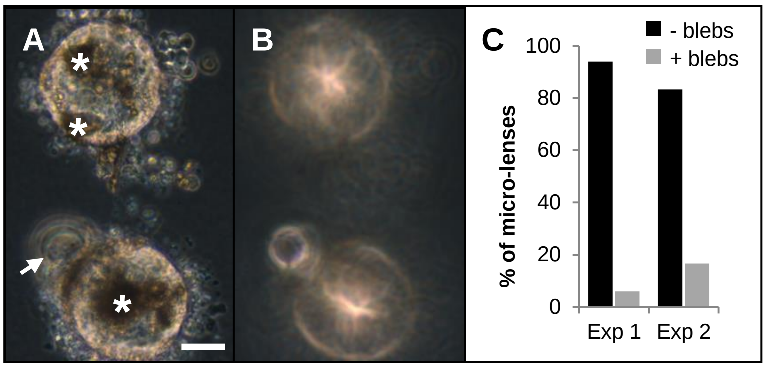

Supplementary Figure S4. Occasional micro-lens features. (A-C) Light microscopy data showing clusters of non-transparent cells (A, asterisks), adjacent to the periphery of two micro-lenses, that did not preclude assessment of light-transmitting regions (A) or focusing ability (B). The presence of ‘bleb’-like structures on some aggregates (A, arrow) had little effect on focusing (B) and were typically relatively infrequent (C). Scale bar: 40 µm.

Development • Supplementary information

Supplementary material Fig. S5

A

1 MDVTIQHPWF KR**TLGPFYPS** **R**LFDQFFGEG LFEYDLLPFL SSTISPYYRQ
51 SLFR**TVLDSG** **ISEVR**SDRDK FVIFLDVK**HF** **SPEDLTVK**VQ DDFVEIHGKH
101 NER**QDDHGYI** **SR**EFHRRYRL PSNVDQSALS CSLSADGMLT FCGPK**IQTGL**
151 **DATHAER**AIP VSR**EEKPTSA** **PSS**

B

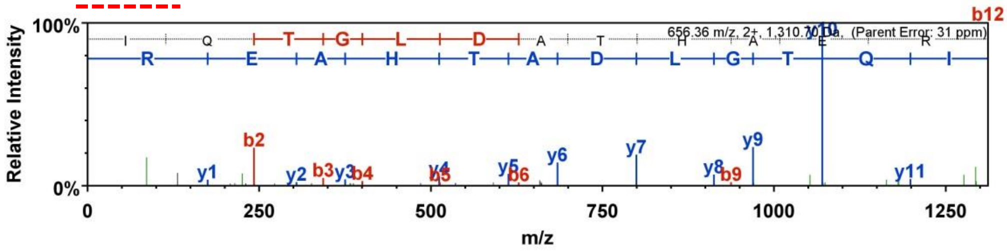

C

1 MSQAAK**ASAS** **ATVAVNPGPD** **TK**GK**GAPPAG** **TSPSPGTTLA** **PTTVPITSAK**
51 **AAELPPGNYR** LVVFELENFQ GRR**AEFSGEC** **SNLADR**GFDR VRSIIVSAGP
101 WVAFEQSNFR **GEMFILEK**GE YPRWNTWSSS YRSDRLMSFR PIKMDAQEHK
151 **ISLFEGANFK** GNTIEIQGDD APSLWVYGFS DRVGSVK**VSS** **GTWVGYQYPG**
201 **YR**GYQYLLEP GDFRHWNEWG AFQPQMQSLR RLRDKQWHLE GSFPVLATEP
251 PK

D

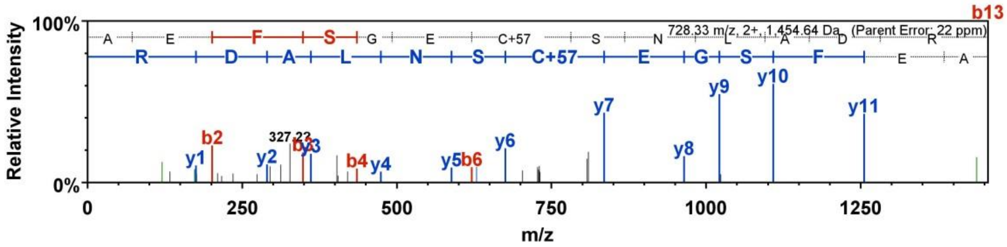

E

1 MTLQCTKSAG PWKMVVWDED GFQGRRHEFT AECPSVLELG FETVRSLKVL
51 SGAWVGFEHA GFQGQQYILE RGEYPSWDAW GGNTAYPAER LTSFRPAACA
101 NHRDSR**LTIF** **EQENFLGK**KG ELSDDYPSLQ AMGWEGNEVG SFHVHSGAWV
151 CSQFPGYRGF QYVLECDHHS GDYKHFREWG SHAPTFQVQS IRRIQQ

F

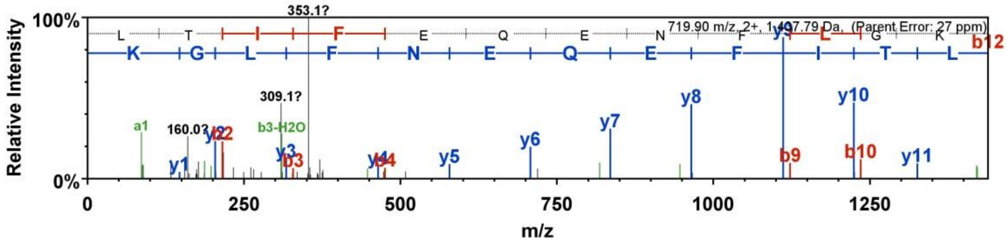

G

1 MASDHQTQAG KPQSLNPKII IFEQENFQGH SHELNGPCPN LKETGVEKAG
51 SVLVQAGPWV GYEQANCKGE QFVFEKGEYP RWDSWTSSRR TDSLSSLRPI
101 KVDSQEHK**II** **LYENPNFTGK** KMEIIDDDVP SFHAHGYQEK VSSVRVQSGT
151 WVGYQYPGYR GLQYLLEKGD YKDSSDFGAP HPQVQSVRRI RDMQWHQRGA
201 FHPSN

H

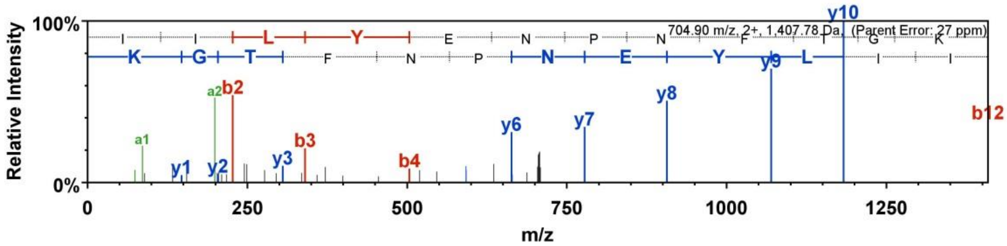

Development • Supplementary information

Supplementary Figure S5. Mass spectrometry analysis of LEC and lens fibre cell crystallins in micro-lenses. (A-H) MS/MS analysis of micro-lenses cultured for 27 days in Stage 3 lens differentiation medium revealed 35% protein coverage of CRYAA (A), 38% coverage of CRYBB1 (C), 6% coverage of CRYBA4 (E) and 5% coverage of CRYBB2 (G). Example raw data peptide identifications are shown for the underlined sequences in CRYAA (A, B), CRYBB1 (C, D), CRYBA4 (E, F) and CRYBB2 (G, H). These data, obtained from 10 pooled micro-lenses, are representative of data obtained from 2 independent micro-lens experiments.

Development • Supplementary information

Supplementary material Fig. S6

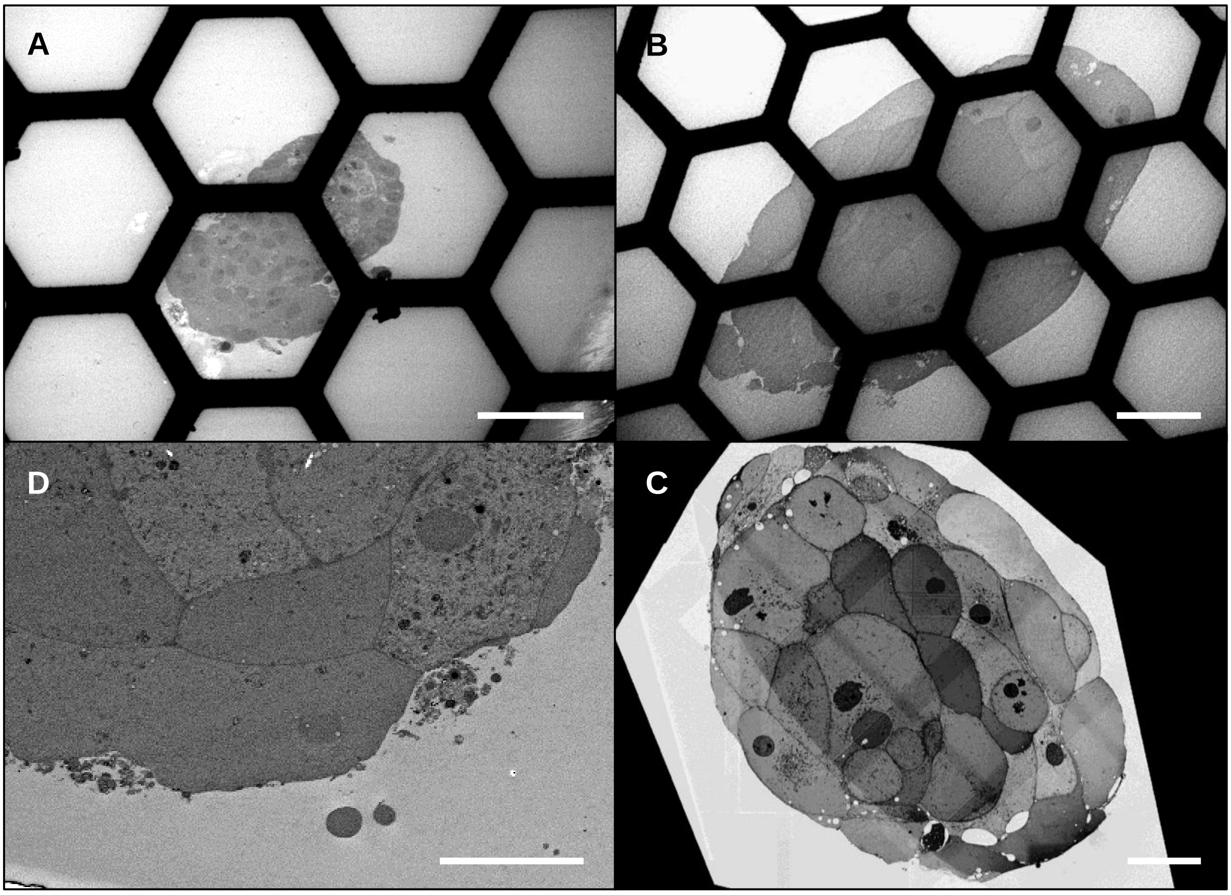

Supplementary material Figure S6. Cellular organisation in ROR1+ cell aggregates. (A-C) Electron microscopy data from an aggregate cultured for 14 days (A) shows the bulk of the tissue consisted of small cells. Scale bar: 50 µm. (B, C) Images of aggregates cultured for 42 days show the bulk of the tissues consist of larger cells with fewer organelles. Scale bars: 50 µm (B) and 10 µm (C, composite image). (D) Lens fibre-like cell adjacent to a LEC-like cell in an aggregate cultured for 24 days, suggestive of rudimentary secondary lens fibre cell differentiation. Scale bar: 10 µm. Images representative of 6 micro-lenses from 2 biological replicates.

Development • Supplementary information

Supplementary material Fig. S7

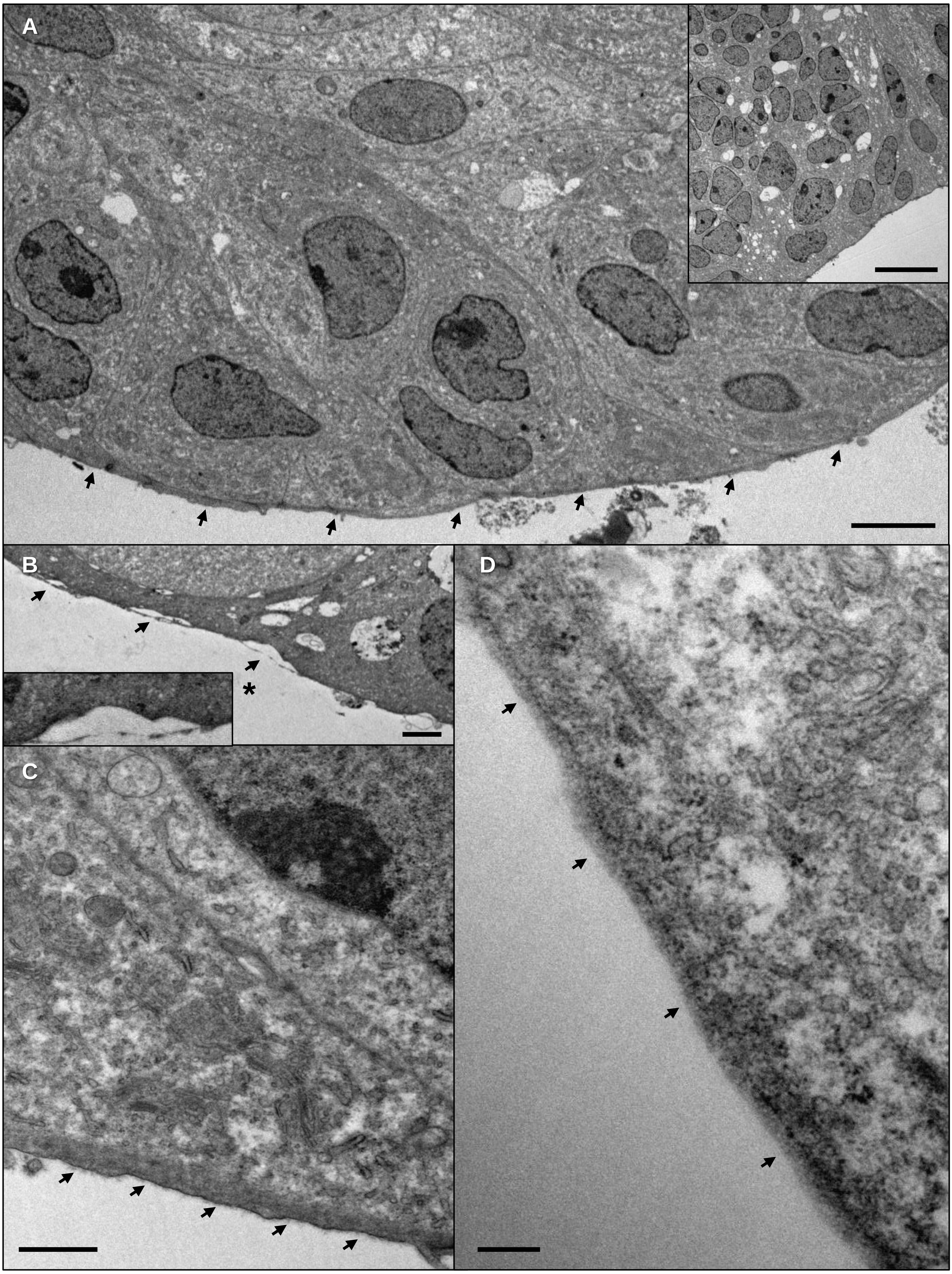

Development • Supplementary information

Supplementary material Figure S7. Lens capsule surrounding ROR1+ cell aggregates. (A-C) Electron microscopy data from a large aggregate (diameter >200 µm) cultured for 42 days. (A) Multilayering of peripheral LEC-like cells adjacent to lens capsule-like material (arrows). Scale bar: 5 µm. Inset: a small area of multilayering adjacent to the peripheral LEC-like cells. Scale bar: 10 µm. (B, C) Lens capsule-like material adjacent to lens fibre-like cells. (B, inset: higher magnification of region indicated by asterisk). Scale bars: 1 µm. (D) Lens capsule-like material (indicated by arrows) adjacent to LEC-like cells. Scale bar: 250 nm.

Development • Supplementary information

Supplementary material Fig. S8

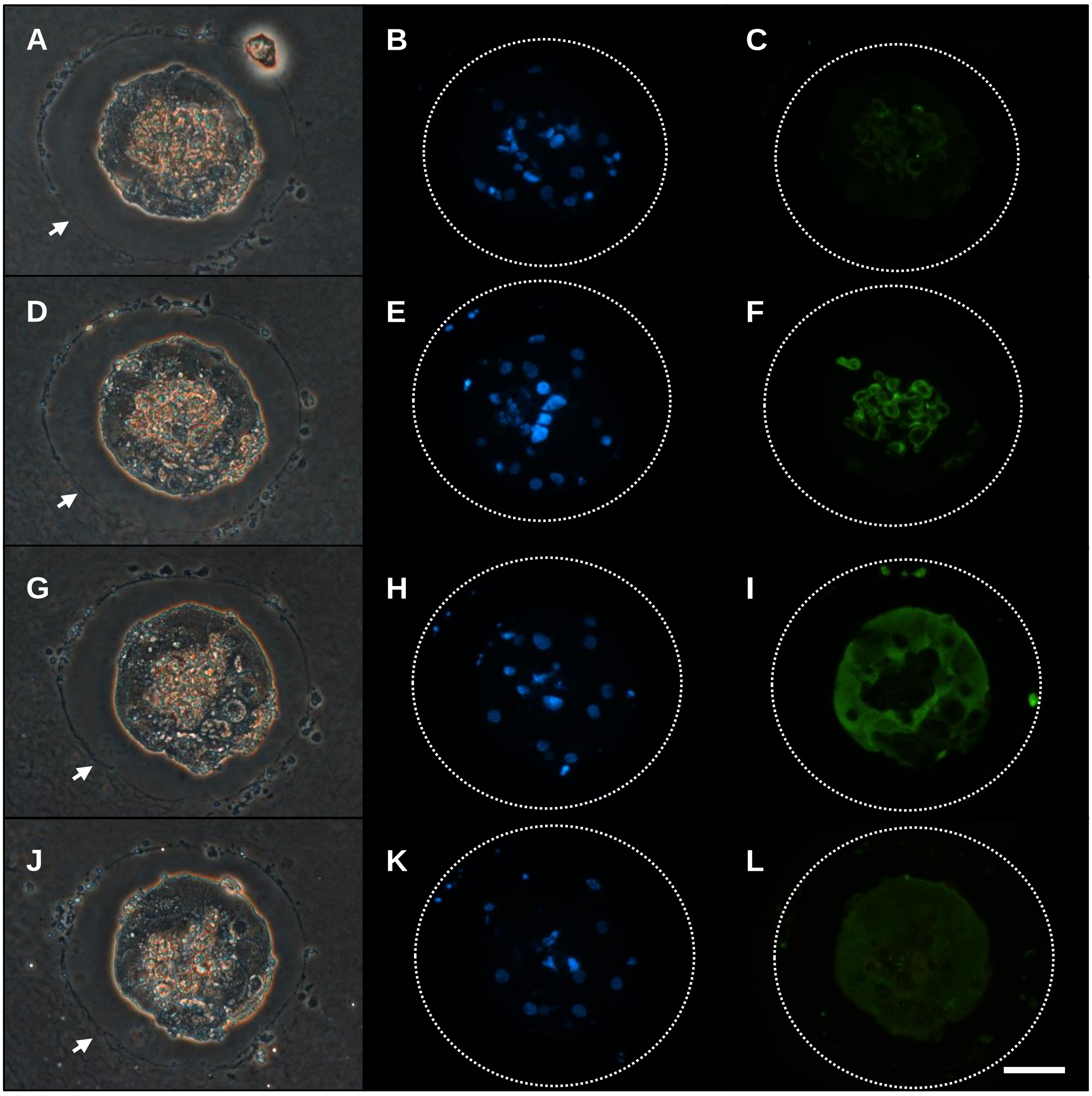

Supplementary material Figure S8. Lens capsule components expressed by LEC-like cells in ROR1+ cell aggregates. (A, D, G, J) Consecutive peripheral sections of a large aggregate (diameter ~200 µm) cultured for 24 days (A is the outermost section). The short fixation time (60 min) permitted detection of laminin and collagen IV (C, F), however, it also resulted in significant shrinkage of the sections that was not observed with longer (24 hour) fixation times. The surrounding agarose used to embed the aggregates during culture is indicated by arrows. (B, E, H, K) DAPI staining of the same sections shown in A, D, G, and J shows the location of nuclei within the fixed micro-lenses. (C, F, I, L) The same sections shown in A, D, G and J after immunofluorescence using anti-laminin (C), anti-collagen IV (F), anti-γ-crystallin (I) and anti-αA-crystallin (L) antibodies. Dotted white lines estimate the original micro-lens boundary. Scale bar: 40 µm. Data representative of 7 micro-lenses from 2 biological replicates.

Supplementary Table S1 and S2. Mass spectrometry analysis of ROR1+ cells cultured in TM17 express LEC but not lens fibre cell crystallins. A list of proteins identified from ROR1+ cells expanded, frozen, thawed and then re-cultured for 6 days in TM17 reveals expression of a- but not b-crystallins. (B) A list of proteins identified from ROR1+ cells expanded, frozen and thawed in TM17 and then cultured for 6 days in Stage 2 lens differentiation medium. These data show expression of a variety of lens fibre cell-specific crystallin proteins.

Click here to Download Table S1-S2

Supplementary Table S3. Mass spectrometry analysis of micro-lenses derived from ROR1+ cells. A list of proteins identified from ROR1+ cell-derived micro-lenses cultured in Stage 3 lens differentiation medium for 27 days shows expression of a-crystallin as well as a variety of lens fibre cell-specific b-crystallin proteins. These data, obtained from 10 pooled micro-lenses, are representative of data obtained from 2 independent micro-lens experiments.

Click here to Download Table S3

Supplementary Table S4. Expression of capsule components and integrins by ROR1+ cells. A list of the most highly expressed integrin, collagen and laminin mRNA transcripts detected in the ROR1+ RNA-seq libraries.

Click here to Download Table S4
